# Dr. Henry F. Campbell

**Published:** 1884-06

**Authors:** 


### DR. HENRY F. CAMPBELL.

It affords us sincere pleasure to notice the elevation of Dr. Henry F. Campbell, of Georgia, to the Presidency of the American Medical Association. This tribute to his genius and attainments is very gratifying to the large body of friends whom his congenial manners and personal magnetism have attracted to him, and to the larger number of admirers who recognize the fidelity, the industry, the ingenuity with which, during his whole professional career, he has cultivated the medical sciences.

While in a sense his election may be regarded as a compliment to the section of his residence, and the kind thought which prompted as well as the graceful manner of bestowing it deserve recognition, there is abundant evidence that the distinction has been conferred upon a highly worthy recipient.

Not alone in one, but in all the departments of medicine, Dr. Campbell has attained eminence; as an anatomist, physiologist, gynecologist, surgeon and physician, he is alike distinguished, and in each the traces of his genius and industry are discoverable in the literature of the period to which he has abundantly contributed. His devotion to his profession has been the characteristic of his life, enthusiasm for the association which has honored him was a part of it, and without being invidious we can truthfully declare *Palmam meruit.*

